# The impact of training and working conditions on junior doctors’ intention to leave clinical practice

**DOI:** 10.1186/1472-6920-14-119

**Published:** 2014-06-18

**Authors:** Christiane Degen, Matthias Weigl, Jürgen Glaser, Jian Li, Peter Angerer

**Affiliations:** 1 Institute of Occupational Medicine and Social Medicine, Centre for Health and Society, Faculty of Medicine, University of Düsseldorf, Düsseldorf, Germany; 2 University Research and Applied Science, German Hospital Institute, Düsseldorf, Germany; 3 Institute and Outpatient Clinic for Occupational, Social and Environmental Medicine, Ludwig-Maximilians-University, Munich, Germany; 4 Institute of Psychology, University of Innsbruck, Innsbruck, Austria

**Keywords:** Intention to leave clinical practice, Postgraduate residency training, Training conditions, Working conditions

## Abstract

**Background:**

The shortage of physicians is an evolving problem throughout the world. In this study we aimed to identify to what extent junior doctors’ training and working conditions determine their intention to leave clinical practice after residency training.

**Methods:**

A prospective cohort study was conducted in 557 junior doctors undergoing residency training in German hospitals. Self-reported specialty training conditions, working conditions and intention to leave clinical practice were measured over three time points. Scales covering training conditions were assessed by structured residency training, professional support, and dealing with lack of knowledge; working conditions were evaluated by work overload, job autonomy and social support, based on the Demand–Control–Support model. Multivariate ordinal logistic regression analyses with random intercept for longitudinal data were applied to determine the odds ratio of having a higher level of intention to leave clinical practice.

**Results:**

In the models that considered training and working conditions separately to predict intention to leave clinical practice we found significant baseline effects and change effects. After modelling training and working conditions simultaneously, we found evidence that the change effect of job autonomy (OR 0.77, p = .005) was associated with intention to leave clinical practice, whereas for the training conditions, only the baseline effects of structured residency training (OR 0.74, p = .017) and dealing with lack of knowledge (OR 0.74, p = .026) predicted intention to leave clinical practice.

**Conclusions:**

Junior doctors undergoing specialty training experience high workload in hospital practice and intense requirements in terms of specialty training. Our study indicates that simultaneously improving working conditions over time and establishing a high standard of specialty training conditions may prevent junior doctors from considering leaving clinical practice after residency training.

## Background

The shortage of physicians is an evolving world-wide problem (e.g. USA [1], Germany [2], Finland [3], Australia [4] and OECD Countries [5]). Although the volume of admissions to medical schools is adequate in most countries to overcome this shortage, not all medical students begin working or spend the duration of their working life in direct patient care [6,7]. A proportion of medical students drops out during medical school, decides to work in a non-clinical field after graduation or even leaves patient care during or after postgraduate residency training [8]. In addition to the resulting physician shortage, the economic cost of education is high if specialist physicians decide to work subsequently in a field other than patient care. Therefore our study addresses junior doctors’ intention to leave clinical practice after residency training.

Adverse working conditions, such as high workload [9] and long working hours [10], are the arguments against a career in medicine repeatedly reported by junior doctors. Specifically, during residency training junior doctors experience a double load: they work in a highly demanding job while being an apprentice in this profession at the same time [11]. This double load may further exacerbate intention to leave clinical practice if unfavourable training and adverse working conditions come together. Furthermore, junior doctors complain about low income compared to hours worked and poor work-life balance [9,10,12]. Although German doctors during and even more after residency belong to high-income classes, specialized doctors can often earn more and find better working conditions in the medical-related industry (e.g., pharmaceutical companies, public administration, or health insurances).

Compared to the evolving problem of physician attrition, research on intention to leave clinical practice or the medical profession is scarce. We found two studies that investigated specifically junior doctors undergoing residency training [2,10]. Moss et al. [10] showed that the most frequently mentioned reasons by 279 UK medical postgraduates who considered leaving medicine belong to working conditions; e.g. poor working environment, high workload, long working hours and poor support within work. Ochsmann [2] reported that among 637 German residents, adverse work characteristics, such as work-related support, availability of postgraduate training possibilities and overtime, increase the risk of thinking about leaving clinical care. Furthermore, Westerman et al. [13] showed for transition from specialty training to a position as hospital consultant that social support facilitates the process through this phase*.* Among later-career doctors, work characteristics also determine intention to leave clinical practice. Among 2,650 Finnish physicians, Heponiemi et al. [14] found that job control reduces intention to leave the profession. In a study of 1,924 French hospital physicians, Estryn-Behar et al. [15] found that intention to leave the profession was positive associated with low quality of teamwork. In conclusion, these studies suggest that physicians’ workplace conditions of adverse job demands, low job control, and low work-related social support are associated with intention to leave clinical practice.

Therefore, in this study, the Job Demand–Control–Support model (JDCS) [16,17] of occupational stress is used as a theoretical framework for the working conditions of junior doctors. JDCS is an extension of Karasek’s original Job Demand–Control (JDC) model [18]. According to the JDCS model, high job demands, low job control and lack of social support at work lead to high work strain, which impairs psychological and job-related well-being. In addition to these well-known organizational stressors we take into account the training conditions experienced by junior doctors. The training conditions used in this study are based on knowledge of standards and learning processes in postgraduate medical education [19-21]. Structure in residency training, support from supervisors and a climate of dealing openly with lack of knowledge are the ideal prerequisites of on-the-job residency training. This study considers intention to leave clinical practice as a measure of junior doctors’ intention to drop out of clinical practice [22,23].

Our study contributes to the current body of research on junior doctors’ intention to leave clinical practice in two ways. Firstly, we aim to identify to what extent junior doctors’ training and working conditions determine their intention to leave clinical practice after residency training. To date, training conditions have been studied insufficiently [24]. Secondly, as adverse working conditions of physicians are well-represented by the established JDCS model, we use the JDCS model as theoretical framework to examine junior doctors’ intention to leave hospital practice. We expect an increased risk of intention to leave clinical practice for junior doctors who have higher working demands, lower control over job activities, lower social support at work and unfavourable training conditions.

## Methods

### Study design

Our analysis is based on a three-wave prospective study of 1,000 residents in Germany. Based on registration data from the Bavarian Chamber of Medical Doctors, all second and third-year residents working in hospitals in Munich and the wider Munich area defined exactly as wide as to cover 1,000 junior doctors were addressed by letter. Pre-coded questionnaires were mailed directly to prospective participants with self-addressed and stamped envelopes. The questionnaires included a composition of validated questionnaires and some additional questions about demographic and workplace characteristics. In Germany, all medical career options depend on residency training that is based in hospital practice. The guidelines for residency training are given by the German Medical Association and define for each speciality the training contents with minimal training duration [25]. The time lag between the surveys was 14 months between t1 and t2 and 19 months between t2 and t3. These intervals were chosen to investigate the junior doctors during their clinical rotation under different training and working conditions. In addition, the last measurement was placed close to the end of residency training, which usually takes between 5 and 6 years to complete. Ethical approval was given by the Committee on Ethics of Ludwig-Maximilians University Munich. Written informed consent was obtained from all study participants.

### Measures

#### Intention to leave clinical practice

The outcome variable was measured with a single item at each time point: ‘Do you want to work in direct patient care after completing your residency in this specialty?’. The Likert-scaled response categories were ‘strongly agree = 1’, ‘agree’, ‘undecided’, ‘disagree’, and ‘strongly disagree = 5’.

#### Working and training conditions

Conditions were assessed using scales from the well-established German self-report version of the Work Analysis Instrument for Hospitals [26-28]. This instrument is specifically designed for work analysis of health care professions in the hospital. *Job autonomy* (7 items) refers to decision-making latitudes and action latitudes at work (e.g. ‘ My work allows for making decisions on which tasks I have to perform’, ‘My work offers discretion on how to do my work’). *Social support* (5 items) describes the quality of relationships with co-workers and supervisors in the department or overall hospital (e.g. ‘In this department there is a trusting relationship between colleagues’, ‘In this department there is a trusting relationship with supervisors). *Work overload* (4 items) refers to work pressure caused by workload and time constrains (e.g. ‘Frequently, there is too much work at once’, ‘Frequently, there is time pressure due to short-term deadlines’). *Structured residency training* (4 items) assesses whether training is based on a training scheme and advancement is defined by the scheme and not dependent on the arbitrary decisions of superiors (e.g. ‘In this department or hospital the training is completed in the intended time of the training curriculum’, ‘In this department or hospital residency training dependents on the arbitrary decisions of supervisors’). *Professional support* (3 items) relates to support and assistance from experienced colleagues in new or challenging clinical situations and medical skill development (e.g. ‘Assistance is available when a difficult work problem cannot be solved alone’ , ‘Adequate supports for developing the skills required to master challenging tasks are often not provided (item reverse-coded)’). *Dealing with lack of knowledge* (3 items) refers to the general opportunity to admit lack of knowledge and skills in front of colleagues and supervisors (e.g. ‘In this department it is possible to admit lack of knowledge or skills to a supervisor’, ‘In this department it is possible to admit lack of knowledge or skills to non-medical staff’).

All items were scored on a 5-point Likert scale, ranging from ‘not at all’ = 1 to ‘yes, absolutely’ = 5. All scales including training and working conditions showed acceptable reliabilities with Cronbach’s alpha coefficients between 0.64 and 0.87 across all measurement waves (details of psychometric information of questionnaire are available on request).

#### Covariates

Year of residency at t1, age (in years), gender (1 = male, 2 = female) and baseline intention to leave clinical practice were adjusted as potential confounders.

### Statistical analyses

A multivariate ordinal logistic regression with random intercept was used to take the ordinal nature of the outcome variable and repeated individual observations into account [29,30]. Odds ratios for the risk of higher level of intention to leave and 95% confidence intervals are reported. Due to the observations nested in individuals, time-varying predictors in the random intercept model represent both the within-individual effect over time and the between-individual effect [31]. Therefore, separate variables for time-varying predictors were included in the analysis to account for differences between doctors and changes within a doctor across the study period; for example, for the time-varying variable ‘structured residency training’, the between-individual effect is predicted by the baseline value of structured residency training, whereas the within-individual effect is predicted by the change of this variable, which is the difference between structured residency training in a given year (t) and baseline structured residency training for each doctor (j) (xjt-xj1) [32]. Measurements at t1 were excluded from the analysis after the calculation of the change variables. Therefore, only individuals with information at t2, t2 *and* t3 or t3 were included in the analysis. Baseline values of constant variables are not lost from the analysis as they are included in each period of the dataset with the same value. The person-period dataset consists of one or two data rows per doctor (1,004 observations in all). An advantage of this applied model is that it allows the estimation of consistent and efficient estimates for unbalanced data structures by using maximum likelihood estimation [30]. The proportional odds assumption of the ordinal logistic regression was assessed with the Brant test [33]. Potential multicollinearity of the between and within-individual effects was assessed by calculating the variance inflation factor (VIF) for all variables. No reason for multicollinearity was found with VIF values of 2.53 and below [34]. To interpret the ORs by the change in odds for a one standard deviation increase in the predictors, all non-dichotomous predictors were standardized (z-transformed).

First, a regression model was calculated for the association between training conditions and intention to leave (Model 1). Second, the association between working conditions and the outcome was estimated (Model 2). The simultaneous prediction of the effects of training and working conditions on intention to leave was assessed in Model 3. Log Likelihood Statistics were used to compare model fit between the models. Additionally, we tested separately for the baseline and change effects, for potential interaction effects between the working conditions and between work overload and the training conditions, in order to test supporting or controlling effects of the training conditions in the presence of work overload. Statistical analyses were performed with Stata 12.

## Results

In the first wave (t1) 621 questionnaires were returned, while 561 and 525 were returned in t2 and t3, respectively. The final sample consisted of 557 doctors (277 men and 280 women), as the analysis of change in the core study variables led to the exclusion of doctors with no follow-up information in t2 and t3, and a number of doctors were excluded because of missing values in core study variables in t2 and t3. All medical specialties involved in patient care were represented; the largest groups at t1 were internal medicine (27.11%), surgical medicine (13.46%), anaesthesia (10.59%), paediatrics (6.82%), general medicine/family medicine (6.10%) and gynaecology (6.10%).

Table 1 shows the frequencies of intention to leave clinical practice (ITL) for the final sample over the three waves.

**Table 1:** Frequencies of intention to leave clinical practice (ITL) for t1, t2 and t3

**ITL**	**t1**		**t2**		**t3**	
	**Freq.**	**%**	**Freq.**	**%**	**Freq.**	**%**
1 = no ITL	315	56.55	276	52.27	235	49.37
2	176	31.60	188	35.61	185	38.87
3	44	7.90	48	9.09	38	7.77
4	17	3.05	14	2.65	9	1.89
5 = high ITL	5	0.90	2	0.38	9	1.89
Total	557	100.00	528	100.00	476	100.00

The 557 doctors under analysis participated at both follow-up time points (447) or at either t2 (81) or t3 (29). Overall, intention to leave clinical practice increased over the study period with more responses for the category ‘high ITL’ and less for ‘no ITL’. The increase in the mean value of ITL between t1 and t3 was significant (Wilcoxon signed-rank test (476) = −2.08, p = .037). Dropout analysis was made for doctors who dropped out from the study after the second wave. Differences in the baseline values of the core study variables for doctors who stayed until t3 (476) and doctors who dropped out after t2 (81) were not found, with the exception of professional support. Doctors who dropped out reported slightly higher values for professional support (two-sided independent t-test (555) = −2.01, p = .045). Additionally, the selection bias was examined for the final sample and for doctors who participated only at the baseline. These doctors (64) differed from the final sample (557) only in their higher baseline value for social support (two-sided independent t-test (619) = −2.44, p = .015).

Descriptive statistics and intercorrelations of the study variables are displayed in Table 2.

**Table 2:** Descriptive statistics and intercorrelations of study variables

		**Mean**	**SD**	**1**	**2**	**3**	**4**	**5**	**6**	**7**	**8**	**9**	**10**	**11**	**12**	**13**	**14**	**15**
1	Intention to leave ti	1.66	0.82															
2	Intention to leave t1	1.59	0.81	.48***														
3	Age t1	30.51	2.64	.04	.02													
4	Residency year t1	2.26	1.17	.00	-.07**	.23***												
5	Structured residency training t1	2.76	0.75	-.13***	-.11***	-.01	-.02											
6	Professional support t1	3.32	0.74	-.10***	-.13***	-.10***	-.08**	.38***										
7	Dealing with lack of knowledge t1	3.77	0.73	-.15***	-.13***	-.07**	-.16***	.21***	.37***									
8	Job autonomy t1	2.75	0.67	-.08***	-.10***	.01	-.00	.26***	.26***	.34***								
9	Social support t1	3.31	0.52	-.13***	-.13***	-.09***	-.08**	.31***	.42***	.40***	.34***							
10	Work overload t1	3.24	0.87	.04	.07**	.05	.13***	-.22***	-.42***	-.25***	-.30***	-.20***						
11	Structured residency training ti-t1	0.03	0.80	-.05	.06**	.01	-.05	-.49***	-.15***	-.05	-.10***	-.15***	.05					
12	Professional support ti-t1	0.03	0.79	-.07**	.02	.04	.06**	-.18***	-.54***	-.11***	-.06*	-.17***	.15***	.39***				
13	Dealing with lack of knowledge ti-t1	−0.09	0.72	-.04	.10***	.01	.03	-.06*	-.16***	-.51***	-.11***	-.12***	.09***	.22***	.30***			
14	Job autonomy ti-t1	−0.14	0.70	-.09***	.02	.05	.03	-.14***	-.15***	-.16***	-.47***	-.15***	.16***	.20***	.23***	.24***		
15	Social support ti-t1	−0.10	0.58	-.07**	.05*	.05	.04	-.10***	-.21***	-.15***	-.12***	-.49***	.11***	.27***	.38***	.38***	.31***	
16	Work overload ti-t1	0.05	0.92	.09***	-.01	-.08**	-.08**	.09***	.16***	.09***	.12***	.08***	-.52***	-.19***	-.31***	-.18***	-.29***	−0.26***

Observations = 1,004; doctors = 557; *p < .05, **p < .01, ***p < .001; overall values; ti with i = 2, 3 and i = 1 baseline.

The change variables showed an improvement for two of the three training conditions. The mean values of structured residency training and professional support increased above baseline values in t2 and t3, whereas, all working conditions experienced deterioration compared to the baseline ratings. The expected theoretical directions of the overall intercorrelations between intention to leave in t2 and t3 and training and working conditions were found. With the exception of baseline work overload, change (from baseline value) of structured residency training (t2 and t3) and change of dealing with lack of knowledge (t2 and t3), all intercorrelations showed low but significant coefficients. Substantial associations between the training and working conditions are also presented in Table 2.

To estimate the prospective impact of training and working conditions on junior doctors’ intention to leave clinical practice we conducted multivariate ordinal logistic regression analyses with random intercepts. The results are presented in Table 3.

**Table 3:** Prospective associations between training and working conditions and intention to leave clinical practice after residency training

	**Model 1**		**Model 2**		**Model 3**	
	**OR**	**(95% CI)**	**OR**	**(95% CI)**	**OR**	**(95% CI)**
**Covariates**						
Intention to leave t1	4.01***	(3.17–5.08)	4.20***	(3.29–5.37)	4.08***	(3.21–5.19)
Age t1	1.03	(0.84–1.26)	1.05	(0.84–1.31)	1.05	(0.85–1.30)
Gender	0.95	(0.65–1.39)	0.95	(0.63–1.43)	0.94	(0.63–1.40)
Year of Residency t1	1.08	(0.89–1.31)	1.14	(0.93–1.40)	1.10	(0.90–1.34)
**Training conditions**						
**baseline training conditions (between effect)**						
Structured residency training t1	0.72**	(0.57–0.92)			0.74*	(0.58–0.95)
Professional support t1	0.95	(0.74–1.23)			1.01	(0.76–1.36)
Dealing with lack of knowledge t1	0.69**	(0.54–0.88)			0.74*	(0.57–0.97)
**change of training conditions (within effect)**						
Structured residency training	0.84*	(0.70–0.99)			0.87	(0.72–1.04)
Professional support	0.82*	(0.68–0.98)			0.91	(0.74–1.10)
Dealing with lack of knowledge	0.76**	(0.64–0.90)			0.84	(0.70–1.01)
**Working conditions**						
**baseline working conditions (between effect)**						
Job autonomy t1			0.82	(0.64–1.04)	0.89	(0.70–1.14)
Social support t1			0.70**	(0.55–0.90)	0.85	(0.65–1.11)
Work overload t1			1.11	(0.86–1.42)	1.02	(0.78–1.33)
**change of working conditions (within effect)**						
Job autonomy			0.75**	(0.62–0.90)	0.77**	(0.64–0.92)
Social support			0.76**	(0.64–0.91)	0.88	(0.73–1.07)
Work overload			1.25*	(1.05–1.50)	1.17	(0.98–1.41)
Log Likelihood (LL)	−889.2		−887.4		−879.4	
Akaike information criterion (AIC)	1808.4		1804.8		1800.8	

Observations = 1004; doctors = 557; 95% CI = 95% confidence interval; OR = odds ratio; *p < .05, **p < .01, ***p < .001.

In terms of overall model comparison, Model 3 was superior on the basis of the log likelihood and Akaike information criterion. The likelihood ratio test for comparing the model fit of Model 3 with the nested Models 1 ($\chi^{2}$(6) = 19.58, p = .003) and 2 ($\chi^{2}$(6) = 16.03, p = .014) confirmed this result. This supports our assumption that the joint analysis of working and training conditions in postgraduate training enables the best prediction of junior doctors’ intention to leave.

Therefore, the regression results of Model 3 were just described in detail in the following. The joint inclusion of both workplace domains provides a different picture compared to Models 1 and 2. Changing training conditions over study time had no impact on doctors’ intention to leave; however, higher-rated structured residency training (baseline value) and higher values for dealing with lack of knowledge (baseline value) decreased the odds of higher levels of intention to leave by a factor of 0.74 (p = .017) and 0.74 (p = .026), respectively. In contrast, changing working conditions over time had an impact on a doctors’ intention to leave. An increase in job autonomy reduced the odds of a higher level of intention to leave clinical practice by 0.77 (p = .005). The baseline level of the working conditions was not associated with intention to leave. Junior doctors’ ITL was highly associated with their initial intention to leave (OR 4.08, p = .000).

In line with the assumptions of the JDCS model, additional analyses were conducted to investigate potential interaction effects of work and training conditions. Furthermore, the inclusion of the three-way interaction (work overload × job autonomy × professional support) yielded some evidence for interaction effects in the analysis for the baseline and change effects. However, after applying the Bonferroni correction to adjust p-values for multiple testing, none of the interaction effects remained significant.

## Discussion

The aim of this prospective study was to investigate the risk factors for junior doctors’ intention to leave clinical practice after specialty training. Our study contributes particularly to research through investigating the joint impact of on-the-job training and working in direct patient care. In the simultaneous model, training and work conditions show different effects on junior doctors’ intention to leave. In regard to working conditions, improvements in autonomy over time may reduce a junior doctor’s intention to leave clinical practice, whereas for the training conditions, a higher training standard in the first years of training may prepares the ground for a lower intention to leave clinical practice.

A potential explanation for the different effects of training and working conditions on intention to leave can be found by looking at the organization of residency training. Residency training in Germany, as in other countries [35], is organized as an apprenticeship with rotating assignments through different clinical departments and specialties. During this period, junior doctors experience various working conditions depending on the work organization and workload in the respective clinical department. Job autonomy covers the aspect of job control and describes junior doctors’ freedom in the arrangement of their own working process [11,36]. In clinical rotation, job autonomy depends on the working processes and decision latitudes in the actual department. Furthermore, the job autonomy of junior doctors may be limited at the beginning of residency but increases once they gain more experience. Therefore, an increase in job autonomy during residency training will be expected by senior residents with a certain level of medical knowledge and skills. In line with our study, Heponiemi et al. [14] indicated that high job control, as measured by decision authority, was associated with a lower level of intention to leave the profession in a random sample of Finnish physicians (2,650 physicians). Furthermore, job autonomy in physician workplaces has been found to have positive effects on general psychological (e.g. psychiatric distress [37], depressive symptoms [38]) and job-related well-being (e.g. job dissatisfaction [37], emotional exhaustion [39]).

In regard to training conditions, it is an overall high standard of the training conditions as opposed to advancement in the conditions that appeared to be associated with lowered intention to resign from clinical practice. In our study sample, junior doctors in hospitals with higher standards of specialty training reported less ITL. In the context of clinical rotation structured residency training and dealing with lack of knowledge are influenced less by workload or work organization; these training conditions are rather an underlying prerequisite of successful on-the-job training. Structured residency training encompasses the existence of and compliance with a training schedule that guides clinical practice and is adhered to by supervisors. Poorly structured residency training is often criticized by junior doctors and can lead to an extension of the residency duration [9,12]. Dealing with lack of knowledge is important for postgraduate junior doctors within training, as working and learning at the same time requires a learning environment in which it is feasible to ask questions openly and admit lack of knowledge or skills [40,41]. In addition, this training condition is to a large extent independent of the working organization and workload of a department, as it describes a supportive learning environment in which it is possible to talk openly to supervisors and medical colleagues.

In terms of our underlying theoretical model, the significant effect of working conditions (in the final model 3) refers to the control dimension of the JDCS model. The fact that we did not find consistent evidence of additive and interactive effects of the JDCS model is in line with previous longitudinal research. De Lange et al. [42] found in their review that of 16 high-quality longitudinal studies examining the JDCS model, only 3 provided support for joint effects of all DCS dimensions.

As junior doctors have different training and working conditions in different countries the generalizability of our findings needs to be considered for each country. We assume larger potential for generalization for countries where training and working conditions are comparable to Germany (e.g., Austria, Switzerland). In other countries generalizability might be partly limited. For example, in the US similar training routines for residents occur like clinical rotation [35]. However, working demands are different since residents in the US frequently work more than 70 hours per week [43] whereas German residents work less than 50 hours per week. Due to the European Working Time Directive working conditions will thus be in general less diverse within European countries.

### Strengths and limitations

The longitudinal research design with three data collection waves is a particular strength of the study. This design provides empirically based insights how changes in the training and working conditions affect intention to leave. Our simultaneous analysis of junior doctors’ work and training conditions takes specific account of the different and joint demands of specialty training. The study therefore reflects the actual working situation of junior doctors in direct patient care and training. A further strength refers to the study population coming from all medical specialties involved in hospital care and private practice.

Our dataset provides no information about non-response after t2. Thus, we cannot infer potential reasons why doctors dropped out of further assessments. Because maximum likelihood estimates were used, this missing information should not introduce inconsistent estimation results like over- or underestimation, as long as the doctors’ dropouts depend on covariates observed in t1 or t2 [30,44]. Therefore, the risk that missing information introduces inconsistent estimation is strongly reduced. In fact, the maximum likelihood estimation is a particular strength of the study as more dataset information could be used in the prospective analysis.

Although a validated standard questionnaire was used to obtain information on training and working conditions and intention to leave all data were based on self-reports. Therefore, common method bias may be a problem for estimating the true associations of the predictor and outcome variables [45].

A potential limitation may be the use of a single-item measure for the outcome variable. Many studies investigating intention to leave use single-item measures, such as thinking about giving up clinical practice [2] or intention to change profession [14]. To our knowledge, no validation study comparing single-item measures and multiple-item measures of intention to leave has yet been conducted. As the construct used to measure intention to leave clinical practice in this study is homogeneous and leaves very little scope for interpretation, we believe that a single-item measure is sufficient in this context [46].

Personal characteristics are not modelled within the JDCS model. Interactions of job stress and personality (e.g. locus of control [47]) have been studied and may be also relevant in our study context. As personal characteristics can´t be influenced by supervisors or managers, we relied on the training and working conditions were a potential improvement may convince junior doctors to stay in the direct patient care environment after residency training. Further research is needed to clarify the role of dispositional characteristics (e.g. self-efficacy, resilience) of junior doctors in the examined relationship.

Another limitation worth discussing is that intention to leave clinical practice may differ from the actual behaviour of leaving. An recent investigation of 1,174 UK family physicians aged 50 or below confirmed that intention to leave direct patient care is a significant predictor of actual leaving [48]. This study used data on doctors’ intention to leave direct patient care within 5 years and followed-up the physicians over the next 5 years to assess the actual leaving behaviour. A former investigation found that intention to leave clinical practice is not an accurate predictor of actual leaving behaviour [49]. However, the study design was criticized because time lags in updating practicing status in the data source (the Physician Masterfile) took much longer than the time span investigated [50]. Drawing on the sound result of the family physician study we think that intention to leave is a valuable predictor of actual leaving.

### Implications

Our study indicates that both improving job autonomy and establishing a high standard of specialty training conditions may prevent junior doctors’ intention to leave clinical practice after residency training. The improvement of job autonomy during residency training could be established via a continuous process in order to reflect the clinical experience that junior doctors have obtained. This may be achieved by permitting experienced junior doctors to use their decision-making scope in the organization of work processes and the application of work techniques. Structured schedules in specialty training seem to be essential for junior doctors in order to achieve training objectives without hurdles and within an appropriate time frame. This may be supported by the implementation of a training plan, the use of training log books and/or the organization of rotation and assignment schedules [51]. Openly dealing with lack of knowledge in a department reflects an underlying learning climate that may facilitates junior doctors learning on the job and would be part of the organizational culture of a hospital. Moreover, a climate of dealing with lack of knowledge openly appeared to give junior doctors security in clinical practice and may also have a positive effect on patient safety [11].

## Conclusion

Junior doctors undergoing specialty training experience high workload in hospital practice, as well as intense requirements in terms of residency training. The findings suggest that training and working conditions should be considered simultaneously when investigating junior doctors’ intention to leave clinical practice after residency training. Our results may indicates that junior doctors have greater intention to leave if they have a lower increase in autonomy in their work, if they are in a less structured residency program and if they cannot openly admit to or discuss their lack of knowledge. Interventions in residency training to reduce intention to leave clinical practice may therefore take these factors into account.

## Competing interests

The authors declare that they have no competing interests.

## Authors’ contributions

CD contributed to the research model, analysis and interpretation of the data. She drafted the article and revised it in concordance with the suggestions of the other authors. PA contributed to the study conception and design and the acquisition and interpretation of the data. MW contributed to the study conception and design and the acquisition and interpretation of the data. JG contributed to the study conception and design and the acquisition of the data. JL contributed to the research model and interpretation of the data. All authors contributed to the critical revision of the manuscript and approved the final version for publication.

## Pre-publication history

The pre-publication history for this paper can be accessed here:

http://www.biomedcentral.com/1472-6920/14/119/prepub
